# Cardiac Arrhythmia and Geomagnetic Activity

**Published:** 2006-01-01

**Authors:** E Stoupel

**Affiliations:** Division of Cardiology, Rabin Medical Center, Petah Tiqwa, Israel

**Keywords:** sudden  cardiac death, cardiac arrhythmia, geomagnetic activity, proton flux, cosmic ray

## Abstract

**Background:**

The purpose of this  paper is a review of  a number of studies considering links between life threatening cardiac arrhythmias, sudden cardiac death (SCD) and the level of environmental physical activity factors like geomagnetic activity (GMA) and opposite them cosmic ray and high energy proton flux. This is a part of studies in the field named Clinical Cosmobiology.

**Methods:**

Temporal distribution of cardiac arrhythmias and SCD daily and monthly were compared to the level of GMA, space proton flux, cosmic ray activity according to neutron activity (impulse/min) on the earth's surface. The cosmophysical data was obtained from the cosmic science institutions in the USA, Russia and Finland (cosmic ray data, partially).

**Results:**

As it follows from the results of  the quoted studies there is an inverse relationship between the frequency of cardiac arrhythmic events and SCD and the level of daily GMA.

**Conclusion:**

Now studies are in progress considering the role of neutron (cosmic ray) activity in the natural history of the mentioned events. According to the various studies, we can presume that the GMA has some protective effect on  cardiac arrhythmias and SCD.

## Introduction

Geomagnetic activity (GMA) is part of the physical environment surrounding us. In last century many studies were published discussing different links between the level of GMA and human homeostasis. They include studies on cardiac arrhythmia and sudden cardiac death (SCD). The medical community is not equivocal  considering those findings. In an editorial comment introducing one recent paper in this field the Editor-in-Chief of “PACE” wrote: “…The question is whether the findings represent a new area for investigation or is a “straw man” [1]. In this brief review some data will be presented demonstrating some links between cardiac arrhythmic events and level of GMA.

## Geomagnetic activity

The geomagnetic field is a physical phenomenon resulting from different rotation speeds of different layers of our planet . The level of activity of the field can be affected by “geoeffective” parts of magnetic fields coming as a result of solar explosions and accompanied giant magnetic fields. A small portion of them can “disturb” the original geomagnetic field, resulting in active or stormy levels of GMA. The level of GMA is measured in Nanotesla every three hours (8 parameters in 24 hours). The six highest results (in K or integrated A  indices of GMA) describe the day as Quiet (I), Unsettled (II), Active (III) or Stormy (IV) day of GMA (Table 1). In  the last decades many changes in the human hemeostasis related parameters were analyzed in relation with the level of GMA [2-4]. But the level of GMA is not only a factor that can be related with some pathologic effects. The field is also a  shield  defending our planet from very energetic and potentially harmful space physical activity ingradients, such as cosmic rays and very closely related to them, high energy space proton flux, that can be more active on the surface of our planet when the GMA is extremely low [5-7]. For more information special cosmophysical literature must be used, or at least the Glossary of Solar-Terrestrial Terms [8].

## Summary of clinical data

### Forensic medicine data

Sudden cardiac deaths (n=43) with signs of coronary atherosclerosis, but without  acute myocardial infarction occurred more often on days of lowest (Quiet-Io) GMA compared with higher levels of GMA.( p>0.001) [2].

### Holter monitoring data

Hourly atrial premature complex (APC) and ventricular premature complex (VPC) number  was more on days of lowest GMA [9,10].

Sudden deaths (n=480) in three hours after beginning of symptoms (before admission, or at the admission department) were higher on days of lowest GMA (p <0.01)[9]. Number of sudden deaths dropped on days of severe geomagnetic storm (very high GMA) in July 2000 - the "Bastille day event" [11]. Arrival of patients to the emergency department  at  a  tertiary university  hospital of patients with atrial fibrillation of new origin (n=653, 1185 days of observation) was inverse correlated with four levels of GMA (r=-0.97, p=0.02) [12].

Ventricular tachycardia episodes by  ambulatory  Holter  monitoring  (n=3019) registered  27% on days of  low (Io-IIo)  GMA and 17% on days of  two higher levels (chi^2^=7.3, p=0.006) [13]. In patients admitted for acute myocardial infarction (n=14,529; 8586 men) cardiac arrhythmic events (PAF, AT,VT, VF, n=2024) were relatively  higher on days of lowest GMA [14]. SCD that were occurring in one hour after the onset of symptoms (n=261) was more on days of lowest GMA in men < 65 years (p=0.06) and women > 65years (n=0.027). SCD in woman younger than 65 years was relatively rare [15].

Discharges (n=402) in 137 days  of 25 patients with implanted ICD for ischemic cardiomyopathy, with impaired left ventricular systolic function,  for VT , VF  were inverse correlated with the level of  four daily levels of GMA, (r=-0.96-0.97,p=0.03-0.04) [16].

The number of SCD (n=516), according to the first aid  service data, was significantly correlated with monthly flux of high energy space protons (>90MeV) - a physical parameter closely related  to cosmic ray  activity (described  according to neutron monitoring data on the earth's surface in impulse/min) and inverse related to the levels of  solar and GMA [17].

A new experimental study in pigs has shown that in very low magnetic fields  a prolongation of QT interval and changes in P and T waves were registered. Those changes  are explained as a result of Calcium ion efflux from the myocytes  following  channel blocking or inactivation [18]. Also, a possibility was discussed that the electrons that are involved in the electrical activity of the heart, are changing in different levels of magnetic field activity - less active in higher magnetic field [19].

Now  in progress  are a number of studies considering the possible role of cosmic ray activity - a factor inverse related to GMA, measured by neutron activity on the earth’s surface in the pathogenesis of life threatening  cardiac arrhythmias and SCD [20,21].

The purpose of this short review is to draw attention on possible environmental physical effects that can be an additional pathogenetic factor affecting  the time distribution of events related to cardiac arrhythmia and sudden cardiac death.

## Conclusion

The presented data, a part of science named Clinical Cosmobiology, has shown that  temporal links exist between cardiac arrhythmia, including life threatening events,  SCD and level of cosmophysical activity. Atrial fibrillation,ventricular tachycardia/fibrillation and SCD are occurring in inverse relationship to the level of GMA. It is possible that the level of GMA is a factor preventing SCD, especially in patients with damaged heart muscle. The role of factors becoming more active in low GMA, like neutron activity, are an object of further studies.

## Figures and Tables

**Table 1:**
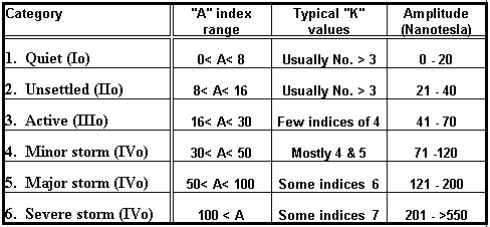
Geomagnetic activity gradation
